# A Case of Traumatic Brachial Neuralgia Treated by Excision of the Cords Which Go to Form the Brachial Plexus

**Published:** 1873-09

**Authors:** Henry B. Sands

**Affiliations:** Surgeon to Bellevue Hospital, and E. C. Seguin, M.D., Physician to the Presbyterian Hospital


*A Case of Traumatic Brachial Neuralgia Treated by Excision of the Cords which go to form the Brachial Plexus.* By Henry B. Sands, M.D., Surgeon to Bellevue Hospital, and E. C. Seguin, M.D., Physician to the Presbyterian Hospital.

We offer the following case, believing it to be unique in its causation, and in the means used to relieve the terrible suffering caused by the nerve lesion.

*History of the Case.*—E. McA., an American, aged 18 years, was wounded in the following manner, at Worcester, Mass. On the fourth of July, 1871, he was aiding in firing a salute with a brass cannon. While he was ramming home the charge, standing on the right of the piece, his left hand by his side, and his right hand driving in the rammer, the piece was prematurely discharged. He was thrown a considerable distance (" 20 feet "?), and lost consciousness. In five minutes consciousness returned, and an examination showed no injury of any part excepting the right upper extremity, which exhibited a badly lacerated wound of the thumb and hand, a fracture of both bones of the forearm in the lower part of its middle third, and an extensive burn of the same part. Patient states in the most positive manner that his hand was absolutely without sensation at the time he regained consciousness, and remained " dead." Very shortly after the accident, the metacarpal bone of the thumb was disarticulated, and as careful a dressing made of the fractured forearm as was allowed by the extensive burn. He was under the care of Dr. Albert Wood.

All apparently went on well until about three weeks after the accident, at which time pain showed itself in the range of distribution of the ulnar nerve of the injured side. In a few days this pain became constant, and assumed an extreme character, extending to the thenar eminence, and affecting the minimus and annulus fingers severely, the medius moderately: the pain was a cutting and tearing one. From that period neuralgia has been the predominant symptom, depriving the patient of rest, exhausting him physically, and quite breaking down his moral tone. Meanwhile, the wound resulting from the amputation of the thumb had partially healed, but no union had taken place between the fractured bones.

On the eleventh of August the patient was brought to this city, and was attended by Dr. Salvatore Caro. Under this gentleman's care, narcotics, morphine, chloral, chloroform, were judiciously administered, but the controlling effect of these drugs was very fugitive; the moment that the patient awoke from artificially produced sleep, the neuralgic pain in the hand and fingers reappeared with extreme violence, and caused the sufferer to groan and shriek. The wound caused by amputating the thumb rapidly healed, and the burn likewise cicatrized in greater part. From day to day the pain seemed, however, to grow worse, and the patient's strength and moral tone to fail. He became so irritable that the dressing of the wounds was a most difficult matter, inasmuch as he thought movement of the shoulder and arm increased his suffering.

On the 14th of August, Dr. Caro called Dr. Sands in consultation, and the other author of this report was allowed by the courtesy of these gentlemen to take part in the examination. The following memorandum is a copy of notes taken shortly after the consultation by Dr. Seguin.

The patient is a well-built, muscular man, much emaciated. During the examination he exhibits a truly extraordinarily nervous state, which his friends declare is quite unlike his usual manner. The face exhibits the traces of severe suffering and broken sleep. The right upper extremity is the seat of slight œdema. In the lower middle third of the forearm is a false joint, caused by the non-union of the fractured ulna and radius, the ends of which are considerably displaced. Nothing remains of the burn except a granulating surface, perhaps one inch in diameter, on the inner surface of the lower third of the forearm. The amputation at the thumb-joint has left a small healthy oval ulcer. The entire upper extremity is motionless upon a pillow, the elbow and forearm being loosely supported by a simple felt gutter splint. Patient fears that the examination will increase the intense shooting, burning, and tearing pain which affects the fingers and the hand; he dreads contact, active and passive motion. Consequently we are surprised to discover that (*a*) patient can make no voluntary motion of any part of the right upper extremity, except slightly raising the scapula, and that (*b*) sensibility is completely abolished as high as the upper part of the arm. The limit between absolute anæsthesia and sensibility is an irregular line which externally rises as high as the point of insertion of the deltoid muscle, and extends several inches lower upon the inner and posterior aspect of the arm. Above this irregular line of demarcation, about the scapular and clavicular regions, there exists great hyperalgesia, the patient complaining that the pain in the hand is excited by slight contact, and shouting and swearing from extreme agony when the scapula is handled. It is remarkable that bringing the scapula forward and holding it in this position gives the sufferer much relief. As regards the degree of anæsthesia existing below the above specified line,—*i. e.*, in lower part of arm, entire forearm and hand,—it may be stated that simple contact is not perceived; that the fracture may be freely handled without causing pain; that pushing pins deeply into the tissues is unnoticed; and that heated objects are not perceived.

From the patient's assertions about the effects of motion and contact upon the neuralgic pain, the belief had grown up that there might exist a relation between the symptoms of nerve injury and the fracture of the bones of the forearm; in other words, it was feared that the great nerves which pass among the muscles of the forearm to supply the hand were caught between the fractured bones, or were being compressed or irritated by fragments of bone. The question to be decided by the consultants, therefore, was, the desirability of cutting down upon the fracture and determining whether any such pathological state as that above stated really existed. Of course, the discovery of the extensive paralysis and anæsthesia above referred to changed the aspect of the case materially. It was evident that we had to deal with an injury much higher up than the fracture, one producing a complete interruption of centripetal and centrifugal conduction in all the nerve-trunks which supply the upper limb. Of course, this being admitted as probable by all present, the conclusion was arrived at that no operation on the distal side of the injury, could relieve the patient of his neuralgia; said neuralgia being a pain referred to the distribution of certain nerves, in accordance with the well-known physiological law of reference of sensations,—a pain whose cause was a nerve-lesion situated in the axillary space, if not higher.

Another consultation (Drs. Caro, Sands, Stephen Rogers, and E. C. Seguin) was held on August 15th, when the question of relieving the suffering of the patient was brought up. Dr. Rogers advised, with the view of interrupting the neuralgia, complete chloroform anæsthesia for a period of twelve hours. Dr. Seguin, considering the neuralgia as dependent upon the irritation of central ends of the injured (ruptured) nerves by newly formed (by repair) connective tissue, recommended counter-irritation to be applied near the supposed seat of nerve lesion, *i. e.*, above and below the clavicle.

On the 28th of August, Drs. F. H. Hamilton and Seguin were asked by Dr. Caro to see the patient. We find him in much the same state, suffering more, if possible; the pain being mainly of a burning character. The patient's excitement and irritability are such that details upon the state of his sensations are very difficult to obtain. The wound in the thumb has completely healed; but the fracture exhibits no signs of union. A careful examination of the state of sensibility shows that anæsthesia is complete in hand, forearm and lower arm as high as limit indicated above. A new test is employed, viz., wire points connected with the maximum secondary current of a strong induction apparatus. Patient's general condition has somewhat improved. The existence of a severe nerve-lesion high in the axillary region (a rupture probably of all the nerves constituting the brachial plexus) being unanimously regarded as certain, and the chances of reunion of the torn nerve fibre and the regeneration of the peripheral parts of the nerves being looked upon as *nil*, it was proposed by Dr. Hamilton that the arm should be amputated near the limit of anæsthesia. It was thought, (1st), that through the operation, some temporary alleviation of the neuralgia might be obtained, and, (2d), that the patient would be rid of a member that would ever remain palsied and useless, and the care of which would interfere with the taking of exercise and with other means of regaining tone and strength. The proposal was concurred in; and, on the 29th, Dr. Hamilton dexterously removed the arm at about its middle, by the circular method. Very little blood was lost, and the operation was well borne. An experiment was made by Dr. Seguin upon the amputated arm immediately after its separation. A double-cell faradic battery was in readiness. The three great nerves—median, ulnar, and musculo-spinal—were rapidly laid bare at the upper part of the separated arm. They had lost their normal glistening, opaque appearance, and looked dirty and translucent. To these nerves, properly isolated upon glass, both the weakest and strongest possible currents were applied without producing the slightest muscular contraction in the arm. The median and ulnar nerves were laid bare in the lower third of the forearm, examined in the same manner, and with the same negative result. Neurility, therefore, was abolished in these degenerated nerves, and a positive proof was obtained by this experiment of the correctness of the diagnosis of nerve rupture. Contrarily, the muscles in every part of the extremity were found highly excitable, even feeble currents producing contractions. The interossei muscles, which respond least well, are infiltrated with serum, and are flabby and pale. A hasty examination of the arm showed the tendon of extensor carpi radialis torn across at its upper part; no other muscles are injured. Muscles and tendons on ulnar side, opposite fracture, are covered with plastic exudations. The broken ends of the ulna and radius are not much displaced, but exhibit no trace of an attempt at repair. The nerves are in nowise involved in the fracture. The articular surface of the elbow-joint has lost some of its polish, and appears red.

One of us again saw the patient at the end of September. "I * learned that a degree of temporary relief had followed the removal of the limb. During the rest of the day of the operation, only slight pain was complained of, but on the succeeding days it returned with increasing severity, until, a fortnight later, it was as great as ever, perhaps even worse. Patient has now completely lost self-control; he swears frightfully, throws articles of furniture about, races up and down stairs in a five-story house, because of

* Transcript from Dr. Seguin's memoranda.

the intense burning, tearing and shooting pains, which are referred to the hand and fingers. The worst times are in the afternoon and evening. Patient is then in a terrible state of nervous excitement; he twists and squirms in his bed or chair, chews violently upon a handkerchief, and the perspiration pours from him. His language is interrupted by groans, oaths, and gnashing of teeth. Hypodermic injections of morphia—twenty and forty minims—with chloral, temporarily control pain. The appetite and nutrition have remained fair. Another consultation is proposed to be held between Drs. Caro, Hamilton, and myself. I am prepared to advise the section or resection of the nerves which go to form the right brachial plexus, at a point nearest the intervertebral foramina. The necessity for the performance of such an operation I base upon the diagnosis of injury (rupture) of the brachial plexus in the region where it is bound down to the vessels. I intend to cut the affected nerves above the seat of injury, and thus cause cessation of neuralgia.

The proposed consultation was never held. It was decided to try the controlling influence of a disciplined household upon his mental condition; and he was accordingly sent the next day to the private institution for the insane under the charge of Dr. Barstow. The patient, let it be remembered, was absolutely sane; but it was thought that many of the new surroundings into which he would be thrown might strengthen his self-control and will to modify his expressions of agony.

On the 30th of October a brother of the patient called at my office, and stated that the family desired to place the patient wholly in my care; and he inquired what means, if any, remained, which would give a chance of relief from his great suffering. The operation above referred to was explained to him, and it was agreed that the trial should be made.

On the 2d of November the patient returned to town, and I visited him the same evening. He has changed very much for the better, his color having improved and his weight increased. He no longer cries out or swears because of the pain, but sits in a chair or lies in bed writhing, sweating, and chewing a handkerchief. The stump is of very good shape, and very nearly well. The neuralgia is still terrible, consisting mainly of shooting, tearing pains, together with some burning, and a sense of cramp in hand, all pain being referred to the extremity of missing member. Patient has been most judiciously treated by Dr. Barstow. He has had no morphia or chloroform for a month. He has eaten heartily, and has walked about a good deal.

An examination shows that the stump is sensitive, perhaps more so than is normal; the shoulder is much atrophied, and droops; the scapula is rotated by the action of the serratus magnus muscle. There exists some tenderness over nerves above the clavicle. The pain is continuous, with exacerbations in the afternoon, and during bad weather. Besides, he complains of his 'hand feeling drawn up,' and of 'sinews working in the arm.' With exception of constipation, no disturbance of any function is present."

November 5th.—The proposed operation is done by Dr. Sands. Present, Drs. Sands, Caro, Geo. A. Peters, Wm. H. Draper, F. N. Otis, T. T. Sabine, John G. Curtis, McCreery, and E. C. Seguin. Drs. Hamilton and Barstow had been invited to attend, but were unable to come. At 10.40 A. M. chloroform was administered, and anæsthesia continued by means of sulphuric ether. An ┛ shaped incision was made, its long arm extending parallel with the outer border of the right sterno-mastoid muscle, and its shorter arm following the clavicle. The flap was then raised, and the connective tissue, with fibres of the platysma myoides and clavicular portion of sterno-mastoid muscle, divided and turned up. The external jugular vein was turned outward uninjured. Across the exposed triangle a vein larger than the external jugular was met with, apparently in very direct connection with the heart (showing systolic impulse,) and, after being tied with two ligatures, was cut across. A little deeper the nerves were exposed without difficulty. It should be added that the latter steps of this dissection were done without cutting instruments. The connective tissue around the nerves did not separate with normal facility; the nerves constituting the brachial plexus were much matted together, and their dissection was by no means easy; still the first rib was plainly felt at the bottom of the wound, the scaleni were visible, and so was the anterior border of the right trapezius. The fifth, sixth and seventh cervical nerves were cut in a lump, a piece fully a quarter-inch in length being excised; the same being done for a thick double cord, which seemed to represent the eighth cervical and first dorsal nerves. The pieces removed looked badly, and the nerves felt more like tendinous cords than like nerves. The surface of section appeared yellowish, showed hardly any trace of secondary fasciculi; and the neurilemma was unmistakably thickened and injected. More of the nerves, (proximal ends) were taken away, Dr. Sands carrying his knife as near the scaleni as was practicable; but even there the sections exhibited the appearances of neuritis. During the operation no hemorrhage worth naming occurred. The carotid and subclavian arteries were both felt, but the phrenic nerve was not seen. Two or three very small arteries and the above-mentioned vein required ligature. The wound was closed by means of stitches placed a quarter of an inch apart, drainage being allowed at the angle of the wound.

This neuritis was not altogether unlooked-for by us. It may prove to be an inflammation which has ascended from the injured point, and which may be successfully treated afterward. Another possibility is, that the nerves have been cut below the seat of injury, in which case the neuralgia will return and persist.

Patient recovered from anæsthesia with much excitement and delirium; an hypodermic injection of sulphate of morphia (two-thirds of a grain, and one-sixtieth of a grain of atropia) being administered before the ether effects had fairly passed off. After 1 o'clock P. M. he slept three hours. At 6 P. M. he is found rational, and moderately exhausted; pulse 120+, skin moist; has some headache; complains of soreness about shoulder, and of severe numbness in absent right hand, "just as when one's foot is asleep." Is chewing a handkerchief as before operation, though this is perhaps from habit. Ordered broths and a draught composed of dr. ss. bromide of potassium and scr. ij. hydrate of chloral, at 11 P. M.

9th.—A certain degree of pain returned after operation. Is quieted by hypodermic injections of morphia, gr. $\frac{3}{5}$, and atropia, gr. $\frac{1}{60}$. Some surgical fever.

10th.—No change in symptoms; a curious sore has appeared on the left ear. It is a superficial dry eschar, about a quarter of an inch square, on outer border of helix, on a level with tragus. Is this a reflex nervous nutrition disorder? Perspires more on right side than left; right brow wet, left quite dry.

26th.—Marked improvement. Numbness, with much burning, still present. Has taken KI. dr. ss. *per diem*. Has m xv. Magendie's solution of morphia (gr. ss.) at midday, m xx. late at night, under the skin. Wound nearly closed.

Dec. 10.—Gaining. KI. discontinued. Has lately taken quinia sulph. gr. v. twice a day; to be continued. Some dozen small blisters have been applied to various parts of stump and shoulder with benefit. Has had exacerbation in changeable, stormy weather. Cigars have seemed to increase effect of morphia injections.

Jan. 1, 1872.—Last week passed through an attack of pneumonia (left lower lobe); defervescence in less than 48 hours. Neuralgia still severe, but decreasing. More self-control. Continue morphia under skin (m xxv. to xl., in two doses), quinia; and ordered cod-liver oil.

April 1st.—The issue was closed about the middle of March; since has had a succession of blisters applied over stump and chest. Has much improved. Now sleeps in daytime and at night; gives much less expression to pain, although, in bad weather or during a change in the weather, he writhes somewhat, and perspires. The pain is of same character as at time of last note; has much burning; very rarely any tearing or lancinating pain. Hyperæsthesia of skin of stump and chest continues. Fingers are still distinctly felt, and are the seat of most pain; the median and index appear glued together. Has noticed a curious associated sensation; which is, that whenever he squeezes strongly with the left hand he feels as if the absent hand were doing the same thing. There is much atrophy of muscles about right shoulder. The right pupil is smaller than the left;* and he sweats much more on the right than on the left side. General health is excellent, weight being 148 lbs.—greater than ever before. Receives injections at office, m xvii. to m xx. (according to weather) in the morning (10 A. M.) and m xii. to m xv. about 7 P. M.

May 25th.—Since last note has improved in respect to neuralgia. Owing to the fact that he has not taken cod oil for some time, his weight has decreased some twenty pounds. The pain is nearly always burning; very rarely is there any shooting pain. The absent fingers appear to be in the same position as that detailed above. During the past three weeks he has observed more or less burning pain in cicatrix above clavicle; this pain is becoming daily more noticeable. He has also suffered somewhat from end of the stump. Has regularly received hypodermic injections of morphia night and morning, m xvi. and xv. of Magendie's solution (m xv.=gr. ss.) An examination of the stump and shoulder is made to-day. These parts are very much atrophied, the acromion and coracoid processes being quite prominent. The scapula has rotated outward and upward in such a way that the acromion process is raised, the posterior border of the scapula drawn away from the spinous processes of the vertebræ, and the inferior angle made to approach the axilla. There is no tendency to the "wing" deformity; *i. e.*, the serratus magnus muscle is not paralyzed. This one and the muscles raising the scapula (trapezius and levator anguli scapulæ) are the only muscles of the region which have escaped atrophy. Forced chest-expansion is very good on both sides. There exists a lateral spinal curvature in the lower cervical region (convexity toward the injured side), and another in the opposite direction (compensatory or result of pneumonia?) in the lower dorsal region. The end of the stump is very firm and sound; the cicatrix above the clavicle is also in good condition. State of sensibility.—The patient states that he has an extensive surface on the right side that is abnormally sensitive. Light contact and pinching are felt a little less distinctly on this zone than on the corresponding parts of the left side; the æsthesiometer test reveals no difference between the two sides. Cold is perceived a little more distinctly on the left side than on the right. While light contact and pinching are less acutely felt in *the part touched* on the right side, these same irritations (and any others) start the neuralgia with a severity proportionate to the acuteness of the impression. This falsely hyperæsthetic region has the following limits: The entire stump and shoulder; the scapular region, and a little of the back inside of and below the scapula; the axillary region, and the

* This disparity in the state of the pupils was seen very shortly after the operation, but no note made of it.

pectoral region as low as a point one inch below the nipple; the inner anterior limit is along the right outer edge of the sternum up to the supra-sternal notch, where the limit extends quite to the median line, thence taking an oblique course along the anterior edge of the sterno-mastoid muscle, then a little forward so as to include the angle of the jaw and a part of its ramus and body; from the lobule of the ear the line extends backward and downward to the posterior angle of the scapula. The teeth on the right side have been so sensitive that he has not brushed them for months; nor has he been able to comb his whiskers on the same side. We repeat that this abnormal sensitiveness is not a true one, not in the parts touched or pinched, but that irritation of this zone excites the neuralgic pains, these being of the nature of associated sensations. The pupil on the side of the injury and operation is distinctly smaller than that on the sound side (left). The perspiration is more abundant, and appears more quickly upon the right side than upon the left. During the examination the left axilla was moist, but two or three large drops of sweat trickled down the side from the right.

During the last two months the neuralgia has been much less influenced by changes in the weather. It is decided to try applications of the actual cautery to the shoulder and chest. Choice is made of the platinum-tipped cautery, applied at white heat, and in a superficial way (Brown-Sequard's method). Morphia to be continued.

June 22d.—The cautery was applied in all some five times without producing any noticeable relief. Pain is severe, but patient has some hours of sleep, and others of comparative ease, while taking only m xiii. of Magendie's solution night and morning. Neuralgia presents same character, consisting mainly of burning, referred to fingers. These last seem to be in peculiar position above described. Patient is left for the summer under the supervision of Dr. A. Brayton Ball.

December 1st.—Since last note no marked change has occurred. Patient still suffers from much burning and from some lancinating. This is, as of old, referred to the fingers and hand, being felt slightly and seldom in stump or supra-clavicular cicatrix. Hallucination regarding position of fingers continues same. He thinks that he has had more pain in last two months, but this is to be judged in connection with the fact that the morphia has not been increased; takes m xiii. night and morning. Pupils are still unequal. Right side (same parts) still exhibits false hyperalgesia, less marked. Has lately combed whiskers and cleaned teeth on that side. Still perspires more on right side. Right side of neck and other parts have been irritated, and no epileptiform symptoms produced. General health good.

There are a number of points in this history, which, we believe, require more extended consideration.

1. The pathological anatomy of the nerves involved. At the time of the amputation, portions of the three great nerves of the arm—median, ulnar, and musculo-spiral—were removed within two hours after the separation of the limb, and immersed in a weak solution of chromic acid. Two or three weeks later, transverse sections were made of these nerves, and treated in a way to be subsequently described.

During the operation performed by Dr. Sands, on November 5th, pieces were removed from the cervical nerves and from the dorsal nerve which go to form the brachial plexus. These pieces, varying in size from one-quarter to one-third of an inch in length, were cut off as near the scaleni muscles as it was possible to carry the knife. As related in the history of the case, these fragments and the nerve-trunks from which they were taken looked wholly abnormal. The connective tissue surrounding them was hardened and thickened, the nervous cords no longer appeared pearly-white or glistening, and the surfaces of section showed no trace of secondary fasciculi, and no attempt at breaking up into bundles, as are seen when a normal nerve is cut across. These fragments were also immersed in dilute chromic acid, and when they were hardened, transverse sections were cut from them. These sections, and those from the neive of the arm, were stained by neutral carmine solution, and the water removed from them by successive washings in alcohol and absolute alcohol. They were then made transparent by being floated upon oil of cloves, and finally mounted in Canada balsam dissolved in chloroform.

Before proceeding to the description of the alterations presented by these sections, it may be well to give a cursory account of the appearance of a normal nerve section prepared by the same (Clarke's) method. In section of a normal sciatic nerve* seen with a power of 65 diameters, every nerve fibre is exhibited as a little circle, within which is a hyaline mass, and in the midst of this mass a red dot placed a little to one side of the centre in most cases. These parts are the axis cylinder as the central dot, the white substance of Schwann or myeline as the hyaline mass, and the membrane of Schwann as the circle or rounded ovoid. These circles (varying a little in diameter) crowded together constitute the secondary nerve bundles or fasciculi, which are so large that most of them are clearly seen by the unaided eye. Between the nerve fibres is an uniting substance which appears faintly striated; and here and there are stronger bands of connective tissue (trabeculæ) which are united with the connective tissue around the fasciculus. This is shown as a thick ring, apparently made up by the aggregation of nearly parallel fibrillæ. Around each secondary fasciculus of a spinal nerve there is such a sheath

* All spinal nerves present essentially the same appearance.

appearing as a ring in transverse sections; and these sheaths are united among themselves by more or less loose connective tissue. In this loose connective tissue run blood-vessels, arteries and veins of various calibre. There are small additional blood-vessels enclosed in the perifascicular sheath and in the delicate tissue which separates the nerve fibres.

*a.* The changes exhibited by the median, ulnar, and musculo-spiral nerves. To the naked eye sections of these nerves show traces of secondary fasciculi, although the picture is far inferior to that seen in the normal section. Under a power of 65 diameters the connective tissue around the nerve and that between the secondary fasciculi appears moderately increased in quantity and density. The perifascicular sheaths themselves have lost their definite outlines, and merge more into the connective tissue lying round about them. The sections of blood-vessels seem but little changed, and only a few granular (yellow) bodies are seen in the interfascicular tissue, mainly in the neighborhood of the vessels. The great alteration is in the nerve fibres. In the fasciculi very few distinct circles are to be seen, the mass constituting the fasciculi appearing as a confused design made up of fragments of circles heaped one upon the other. In none of the remaining circles can an axis cylinder be satisfactorily recognized. In such circles as subsist, the hyaline substance within them (myeline) appears more refracting than is usual, and is often concentrically striated. We have here the lesions characteristic of the Wallerian degeneration, *i. e.*, disintegration of the nerve-fibres, with proportionately little change in the framework of the nerves.

*b.* The sections from the nerves excised on November 5th. These present an altogether different appearance. To the unaided eye they appear like sections of some dense, indistinctly fibrillated tissue, tendon for example. Under a low magnifying power the general sheath of the nerve is seen very much hypertrophied. The secondary fasciculi vary immensely in size and appearance. A few are still rounded, encircled by a distinct sheath, and fairly filled with nerve fibres in better or worse condition. The majority, however, are broken up into innumerable smaller bundles, the separation being effected by the formation of distinct bands of fibrillated connective tissue in the place of the scanty network described as lying between the fibres in a normal section. Between many of these fragmented fasciculi are huge masses of wavy, dense connective tissue, with abnormally large vessels, and with a great quantity of granular pigment deposit. This yellowish pigment lies principally immediately around the blood-vessels, or in the connective tissue near them.

As regards the nerves themselves, it may be stated in general terms that they are in a state of atrophy. In one fasciculus, for example, there are very few fibres which present the circular outline, hyaline mass, and eccentric dot characteristic of the normal fibre seen in transverse section. The vast majority are much smaller than usual (appearing of about the same dimension with 300 diameters as normal fibres do with 65); they vary immensely in diameter, and many are represented only by parts of small circles. No masses of embryonic cells are seen in any part of the preparations.

To resume: The nerves in the upper cervical region present the lesions characteristic of chronic neuritis, viz., much increase and condensation of the framework, with comparatively minor change in the nerve fibres. In other words, the pathological process in these nerves has been primarily hyperplastic, and the nerve atrophy secondary and incomplete; whereas, in the nerves removed from below the axillary space, the nerve atrophy was complete and primary, the changes in the framework very slight. In one case we have the lesions of chronic hyperplastic neuritis; in the other, those of the Wallerian degeneration.

2. Nature and seat of the injury to the nerves.

The absolute anæsthesia of nearly the whole arm exhibited by the patient previous to its removal, and which probably existed immediately after the infliction of the injury, points to a complete solution of continuity in all the nerves which supply the lower arm, forearm, and hand with sensory filaments.* Further, the patient had complete paralysis of muscles situated far above the limit of anæsthesia, those which act upon the upper part of the humerus and some of those moving the scapula. The distribution of motor palsy and of anæsthesia in this case fully illustrated van der Kolk's law of distribution of sensory and motor filaments of one nerve-trunk, viz., that the former are sent to parts which are moved by muscles innervated by the latter.† We therefore had ample clinical reasons for localizing the injury at least as high as those parts of the brachial plexus which lie behind and just above the clavicle, and also for considering that the injury consisted in a complete disruption of the nerve-trunks. Another possibility presented itself to our minds, viz., the tearing out of the roots of the nerves which constitute the brachial plexus, from their attachment to the anterior and posterior aspects of the spinal cord. Such an accident has been placed on record by Flaubert,‡ occurring as a consequence of forced extension made to reduce an old dislocation; but in this case the patient died in a few days with symptoms

* Compare Mitchell, Injuries of Nerves, p. 227. Philadelphia, 1872.

† Schroeder van der Kolk. On the Minute Structure and Functions of the Spinal Cord and Medulla Oblongata. Translated by the New Sydenham Society. Vol. iv., 1859, pp. 8, 9.

‡ Repertoire General d'Anatomie et de Physiologie Pathologique. Vol. iii., p. 55. Cited by Le Bret, mem. de la Soc. de Biologie. 1853.

of spinal cord inflammation, corroborated by the autopsy. Guided by the result of this case, and by the fact that our patient had at no time presented any symptom of spinal meningitis or myelitis, we felt reasonably certain that the nerve-roots in his case had not been torn out. Having thus excluded intra-spinal rupture, and determined with certainty the lowest possible limit of the injury, the question arose, whether we could arrive at a still more exact knowledge of the seat of nerve rupture; where in this tract between the intervertebral foramina giving issue to the fifth, sixth, seventh, eighth cervical, and to the first dorsal nerves, and the upper limit of the axillary space, was the laceration most likely to have taken place? It appeared to us impossible to make a satisfactory answer to this question. The microscopical examination of the nerves corroborated the diagnosis reached upon clinical grounds, since the sections taken from the upper part of the cervical nerves showed neuritis, while those cut from nerves below the axilla exhibited the changes of descending or Wallerian degeneration. It is therefore right to conclude that the excision has been made as intended, above the seat of laceration.

In this connection it may not be amiss to recall the exact mechanism of the accident. The patient's right hand was firmly clasping the rammer, and all the muscles of the arm were in activity during the effort of ramming home the charge. The explosion naturally drove the hand forward and outward with incredible violence, the arm following the same direction, and being for the moment in a state of extremely violent extension. So enormous was the strain upon this limb that the patient was projected bodily many feet. We see no reason for not admitting that the fracture of the bones of the forearm occurred at the beginning of this movement of extension. This being granted, it follows that a great strain was put upon the soft parts which still connected the lower part of the forearm with the upper, and that the blood-vessels and nerves were greatly elongated. During this elongation the nerves gave way at their weakest point, *i. e.*, where they are most firmly bound down, and where they interlace and anastomose —behind the clavicle.

Besides Flaubert's case above referred to, we have met with quite a number of instances of obscure nerve injury, caused by the reduction of old shoulder dislocations, but the details given are so meagre as to make the cases quite useless. An exception to this statement is the case recorded by Le Bret.* A young soldier, who had dislocated his right shoulder, underwent the operation of reduction on the same day. The traction was done by men pulling upon a sheet firmly tied around the arm just above the elbow. Immediately after the reduction, without any special pain having been felt, the patient noticed that his arm and forearm were paralyzed.

* Memoires de la Soc. de Biologie de Paris, 1853, p. 119.

When seen by Le Bret, five months later, there existed complete anæsthesia below the bend of the elbow, besides palsy of the arm. The corresponding side of the neck had lost motion, and was anæsthetic; the right upper eyelid covered the globe, and vision was impaired; the right iris was slightly contracted. There were some lancinating pains in fingers and arm. The nerves (inner aspect of arm, and above clavicle) were tender to pressure. . Some improvement took place in motion of arm and neck, and the ptosis was cured. The author believes that the nerves were torn across in the region of the brachial plexus.

3. The demonstration of persistent muscular irritability at a considerable period after the muscles had ceased receiving nervous influence.

The arm was removed eight weeks after the reception of the injury, and, as related above, while no muscular contractions could be obtained by faradizing the nervous trunks at various points, almost normal movements were produced by the direct application of the current to the muscles themselves, even those (interossei) which had apparently suffered much in their nutrition. The bearing of this experiment upon the question of the independence of muscular irritability might detain us awhile, were it not that this paper has already reached a considerable length. Let it suffice to state that this result agrees with that obtained in the inferior animals. The fact that functional capacity survives in muscles for a period six or twelve times longer* than in nerves, in cases where those organs have been cut off from communication with the spinal cord, has been demonstrated by a great number of physiologists. Among the earlier of these we may name Marshall Hall, J. Muller, Gunther and Schon; the latter fixing the date of loss of excitability in nerve-trunks at eight days after section. Later experiments by Longet, Schiff, Landry, Vulpian, and many others have resulted in positively limiting the time at four days. On the other hand, these observers agree in stating that muscles retain for much longer period the power of reacting under immediate stimuli. Some of Longet's† conclusions on this point are worth reproducing:

"1. In mammals, a motor nerve, when separated from the cerebro-spinal axis, loses all excitability after the fourth day. At that time the application of mechanical, chemical and electrical irritants to any part of the distal end of the nerves is followed by no muscular contraction.

* Dr. Brown-Sequard asserts that there is sometimes no diminution of muscular irritability: he has found it as great as in the normal state 19 months after the whole central end of the facial nerve has been drawn out from its exit at the stylo-mastoid foramen. Bulletin de la Societe Philomathique, 1847, p. 83.

† Traite de Physiologie, t. ii., p. 619. Paris, 1869.

"2. Contrarily, a muscle whose motor nerve is no longer excitable, will, even after the lapse of twelve or more weeks, respond perfectly to any direct stimulus."

Landry,* however, states that in the human species, muscular irritability under these circumstances is abolished in the seventh week. The almost perfect response of the muscles to stimuli in our case, and their apparently normal structure at the end of eight weeks, completely overthrows Landry's conclusion. The causes of error in the author's observations lay, 1st, in the fact that he was unable to apply the electric current directly to the muscles, although he made use of electro-puncture; and that, 2d, in all likelihood there existed in his cases more or less active impairment of nutrition in the paralyzed muscles, owing to irritation of the nerves at their origin.

Vulpian† rightly insists upon the value of the fact observed by him in animals, that muscles deprived of innervation which do not contract when the electric (faradic?) current is made to pass through the moistened skin, do so fairly when the electrodes are placed immediately upon the muscular substance; and he goes on to throw doubt upon the observations made by clinicians in regard to the early (fourth—eighth days) loss of electro-muscular contractility in certain palsies—the "rheumatic" paralysis of the face, for example. It is to be regretted that in our case the patient's great suffering deterred us from faradizing the muscles of the arm before its amputation.‡ As it stands, our observation is in favor of a prolongation of muscular irritability in man after nerve section for a period quite as long as that determined in the lower animals.

4. Some of the symptoms appear to us especially interesting.

(*a*) In the first place, there are signs pointing to a paresis of the vaso-motor nerves on one side of the face, neck, and chest. The right pupil was noted as smaller than the left immediately after the operation, and from an early period the patient perspired much more upon the right side than the left. Besides, there was a peculiar condition of sensibility on a large extent of the right side of the body. At one period this is spoken of as hyperalgesia; but a later examination showed that there was no abnormal tenderness in the part touched, and that the pain produced by contact was felt in the absent arm and hand. Still, it should be borne in mind that the patient's self-control and estimation of the nature of his sensations were not always normal, so that it cannot be asserted that there did not exist, at an early period, true hyperalgesia. The æsthesiometer certainly taught us nothing. The extensive surface, falsely sensitive, bore the same relation to the

* Traite complet des Paralysies, t. i., pp. 40, 41. Paris, 1859.

† Lecons sur la Physiologie der Systeme Nerveux, p. 245. Paris, 1866.

‡ Through causes beyond our control, the galvanic current could not be applied to the nerves and muscles in the above-detailed experiment.

brachialgia that many "tender points" do to ordinary neuralgia. An impression transmitted to the spinal cord, at a point near the portion which gives origin to the nerves supplying the region affected with neuralgia, causes action of the sensory tract connected with these nerves, and consequently produces a *referred* sensation of pain. One of us* had occasion to observe a curious example of this *associated painful sensation* in his own person, last summer. A lower incisor tooth had become the seat of tartar deposit, and the gum below was shrunken, red, and tender to the brush. There had never been toothache. One day a small pimple appeared on a level with the upper margin of the thyroid cartilage on the same (right) side as the unhealthy gum; and during the entire period of growth and maturity of the pimple, pressure (even light) upon it produced an acute pain in the gum around the above-mentioned tooth. The experiment was repeated scores of times; and it was further observed that touching the gum did not produce pain in the pimple. Here an impression made upon a branch of the superficial cervical plexus, transmitted to the sensory tract of the upper cervical cord and medulla oblongata, excited in the latter action of the cells connected with the third branch of the trigeminus.

(*b*) The disturbance of nutrition, which produced a slough upon the left helix, is difficult of explanation. It is well to remember in this connection that Brown-Sequard produces gangrene of the edges of the external ear, at will, in guinea-pigs, by injuring the medulla oblongata.

(*c*) The burning pain (causalgia of Mitchell) did not appear immediately after the injury; this being in accordance with the rule laid down† by the distinguished author just named. As regards the date of the appearance of this peculiar pain, we can obtain no definite information.

The expressions of agony, in action and words, employed by our patient corresponded singularly with those recorded by Dr. Mitchell in his works upon nerve injury.‡

With reference to the extraordinary severity and persistence of burning pain in cases of injury to nerves, we would recall the fact first distinctly stated by Cruveilhier,§ that loss of the power of perceiving thermal impressions occurs later than the loss of various other varieties of sensibility, and indicates absolute anæsthesia; and we suggest that inasmuch as the thermal sense is the last to disappear in gradual diminution of sensibility, so in a neuralgia caused by irritation of nerve-trunks, this most deeply rooted, or

* Dr. Seguin.

† Mitchell. Injuries of Nerves, and their Consequences, p. 197. Phila., 1872.

‡ Compare also, Mitchell, Morehouse, and Keen. Gun-Shot Wounds and other Injuries of Nerves. Philadelphia, 1864.

§ Anatomie Pathologique, liv. xxxviii., p. 9.

most fundamental mode of sensation is most affected, and burning is felt acutely when common pain and formication have almost or quite ceased. It is well known that extreme irritation of the skin, after producing ordinary pain, causes intense burning; an event frequently met with in surgical practice; and, moreover, the contact of extremely cold bodies with the skin sets up a painful sense of heat.

5. The operation above described is believed to be the only one of its kind ever attempted. Excision or division of the spinal nerves has generally been performed on the smaller branches; and, excepting the case herewith related, has never involved the primary trunks near their points of exit from the spinal canal. Neurotomy, when undertaken for neuralgia of traumatic origin, has, in a great many instances, effected a permanent cure, and in these cases is far more likely to prove successful than when it is performed for the idiopathic forms of the disease. If the nerve-tissue is healthy at the point of section, the operation can hardly fail; yet success has followed the operation in not a few cases where the divided nerve was thickened and inflamed. In the lower extremity, excision of the smaller nerves has repeatedy been performed, and in several instances the great sciatic has been either excised or divided. Dr. Mitchell* reports a case in which Dr. Nott excised an inch and a quarter of the great sciatic nerve, close to its point of exit from the pelvis, for traumatic neuralgia, caused by a gunshot wound of the leg. Amputation of the leg, reamputation of the stump, excision of the sciatic nerve in the popliteal space, and amputation of the thigh, had already been performed in succession without avail. Partial relief is stated to have followed the final operation performed by Dr. Nott.

Other cases of division of the great sciatic nerve are recorded by Malagodi, Mayor, Nelaton, and Jobert de Lamballe. In Jobert's case the operation was performed for sciatica. Pain ceased at once, but death occurred six months subsequently from paralysis and bed-sore.

In the upper extremity, excision of the median and several other branches of the brachial plexus has often been practiced, and with various results.† In some cases the operation has effected a complete and permanent cure; while in others it has afforded no benefit. Several years ago one of the authors‡ treated a patient in Bellevue Hospital who suffered from violent neuralgia and chorea, caused apparently by a neuroma which had formed upon the face of a stump after amputation of the arm near the shoulder-joint. The neuroma was laid bare by dissection, and was found

* S. Weir Mitchell, op. cit., pp. 285, 286.

† Schmidt's Jahrbucher; Bd. cxxxv., p. 220; cxxii., p. 218; Bd. cxiii., p. 298 *et seq.*

‡ Dr. Sands.

to be connected with all the descending cords of the brachial, excepting the circumflex. These were pulled downward, and, together with the axillary vessels, divided at about an inch above their seat of attachment to the neuromatous swelling. The neuralgia was relieved by the operation while the patient remained under observation, but the choreic symptoms persisted. He left the hospital about two months after the operation.

In the case which forms the subject of this article, the operation of excision of the spinal nerves was undertaken partly as a last resort, and partly because it was thought that the danger of performing it would be considerably reduced, in consequence of the previous removal of the arm by amputation. It is interesting to observe, however, that no serious nutritional changes, except those affecting the muscles, took place in the parts supplied by the divided nerve-trunks.

Another point of interest is the practicability of the operation, when considered merely with reference to the difficulty and danger attending its execution. Under ordinary circumstances, supposing the nerves to be healthy near the points of section, the operation would cause no embarrassment to a skillful surgeon, and all the cords of the plexus might be exposed and divided without dangerous interference with the neighboring bloodvessels. But, even in the present cese, where the nerve-trunks were pretty firmly adherent to the surrounding tissues, their isolation was satisfactorily accomplished by careful dissection; and the wound made by the operation healed readily, without profuse suppuration.

Examination of the nerves excised led to the unsatisfactory conclusion that they were diseased above the line of section; and it is not easy to understand, on anatomical grounds, why any benefit should have followed the operation. The nerve-trunks, however, were divided pretty close to the intervertebral foramina; and, if it be assumed that the cause of pain resided in their proximal ends, it is not improbable that the tension of the latter may have been diminished, and their relations otherwise favorably altered as a consequence of the handling to which they were subjected previously to their division. Such an explanation seems plausible, from the results that attended an operation recently performed by Professor von Nussbaum. It may also be supposed that a cutting off of a considerable portion of irritated nerve-trunk from communication with the spinal cord diminished the neuralgia, by reducing the total amount of irritation transmitted to the nervous centre.

We may sum up the case by stating that a neuralgia of a class known to resist all ordinary treatment was much relieved by an operation not dangerous in itself. We did not obtain radical success, because we failed to find healthy nerve-trunks at the place of section. The diagnosis of the seat of injury was correct enough, but the ascending neuritis baffled us.

We are indebted to Dr. Caro for a statement of the case as it appeared to him, but as his letter contains nothing that is not recorded in the above history, we take the liberty of omitting it.—*Archives of Scientific and Practical Medicine.*

